# Associations Between General and Specific Psychopathology Factors and 10-Year Clinically Relevant Outcomes in Adult Swedish Twins and Siblings

**DOI:** 10.1001/jamapsychiatry.2023.1162

**Published:** 2023-05-10

**Authors:** Erik Pettersson, Henrik Larsson, Brian Mathew D’Onofrio, Paul Lichtenstein

**Affiliations:** 1 Department of Medical Epidemiology and Biostatistics, Karolinska Institutet, Stockholm, Sweden; 2 School of Medical Sciences, Örebro University, Örebro, Sweden; 3 Department of Psychological and Brain Sciences, University of Indiana, Bloomington

## Abstract

**Importance:**

General and specific factors of psychopathology are associated with future adverse outcomes, indicating that they might be useful for identifying individuals at greatest risk. However, it remains unknown if these associations are attributable to confounders that may influence both the psychopathology factors and later outcomes.

**Objective:**

To analyze associations between psychopathology factors and clinically relevant outcomes within family pairs, adjusting for unmeasured confounds by applying co-twin control and sibling comparison designs.

**Design, Setting, and Participants:**

This longitudinal cohort study with a follow-up range of 9 to 13 years included all Swedish twins born from 1959 to 1985 who participated in the Study of Twin Adults: Genes and Environment (60% response rate) and the oldest pair of all Swedish siblings born from 1959 to 1985 per the Multi-Generation Register. Twins were evaluated based on responses to a hierarchical factor model derived using multivariate statistics. Sibling pairs were evaluated based on psychiatric diagnoses per the Swedish National Patient Register. Information on outcome events and prescriptions were derived from the National Patient Register, Prescribed Drug Register, and Crime Register. Baseline assessment was in August 2005, and data were analyzed from January 2022 to February 2023.

**Exposures:**

Hierarchical factor model consisting of 1 general and 4 specific factors fit to 48 psychiatric symptoms on which twin participants self-reported in 2005 and 1 general and 3 specific factors fit to 9 register-based psychiatric diagnoses assigned to sibling participants prior to 2005.

**Main Outcomes and Measures:**

Outcomes consisted of 7 register-based events occurring after 2005, including suicidal behavior, substance overdoses, and criminal suspicion or convictions (data available until the end of 2013), and prescription of antidepressants, antialcohol or antiopioid medication, antipsychotics, and stimulants (data available until the end of 2017).

**Results:**

The study included 32 328 twins (mean [SD] age, 34 [8] years; 16 076 [49.73%] male) and 1 942 106 siblings (mean [SD] age, 34 [7] years; 991 500 [51.05%] male). General psychopathology was significantly associated with all 7 outcomes within sibling pairs (mean within-pair odds ratio [OR], 2.28; 95% CI, 2.19-2.37) and dizygotic twin pairs (within-pairs OR, 1.65; 95% CI, 1.38-1.98) and with 3 outcomes within monozygotic twin pairs (mean within-pairs OR, 1.77; 95% CI, 1.35-2.36). Within sibling pairs, the specific internalizing factor was associated with antidepressant prescriptions (within-pairs OR, 1.65; 95% CI, 1.59-1.71), the specific substance misuse factor was associated prescription of antialcohol and antiopioid medication (within-pairs OR, 2.36; 95% CI, 2.20-2.54), and the specific psychotic factor was associated with antipsychotic medications (within-pairs OR, 1.61; 95% CI, 1.51-1.72). Similar results emerged within twin pairs.

**Conclusion and Relevance:**

In this cohort study, general psychopathology was significantly associated with all 7 outcomes within sibling and dizygotic twin pairs and 3 outcomes within monozygotic twin pairs at 10 years. Within twin and sibling pairs, the specific factors were primarily associated with related outcomes. Several of the associations in this cohort study could not be attributed to unmeasured confounds shared by family members, suggesting that interventions toward broad psychopathology dimensions might help reduce the risk of future clinically relevant events.

## Key Points

**Question:**

Are general and specific factors of psychopathology associated with 7 clinically relevant outcomes within adult sibling and twin pairs at 10-year follow-up?

**Findings:**

In this cohort study including 1 974 434 participants, general psychopathology was significantly associated with all 7 outcomes within sibling and dizygotic twin pairs and 3 outcomes within monozygotic twin pairs at 10 years. Within twin and sibling pairs, the specific factors were primarily associated with related outcomes.

**Meaning:**

The findings in this study suggest that associations cannot be attributed to unmeasured shared confounds, and interventions toward broad psychopathology dimensions might lead to a reduction in future clinically relevant events.

## Teaser

This cohort study evaluates the association between psychopathology factors and clinically relevant outcomes at 10 years among twins and siblings.

## Introduction

Patients in psychiatry often present with several diagnoses or symptoms.^1,2^ One way to summarize comorbidity is to estimate a hierarchical factor model, which includes 1 general and several specific psychopathology factors.^3,4^ The general factor captures overlap among all indicators, whereas the specific factors (eg, specific externalizing problems) capture overlap among a subset of indicators (eg, problems with alcohol, drugs, and the legal system) not accounted for by the general factor.

Although hierarchical factor model scores are associated with future clinically relevant outcomes (eg, suicidal behavior), highlighting their usefulness for identifying those at greatest risk,^5,6,7,8,9,10,11,12,13,14^ such associations could arise for at least 2 reasons. First, the associations might be attributable to confounders, which can either be shared by family members (eg, childhood conditions or genetics) or not shared by family members (eg, different groups of friends), that influence both responses to the hierarchical factor model and later outcomes. If this is the case, attempting to intervene on the factors would not be expected to reduce the probability of the outcomes. Second, the hierarchical factor model might be in the causal pathway toward the outcomes. If so, intervening on factors could be expected to reduce the probability of future outcomes.

One way to control unmeasured confounds is to use natural experiments,^15,16,17,18^ such as pairs of siblings or twins. Because they are matched on all unmeasured confounds shared in families, including childhood conditions (eg, parenting styles) and segregating genes (25% for half-siblings, 50% for siblings and dizygotic twins, and 100% for monozygotic twins),^19,20,21^ all family-constant unmeasured confounds are adjusted by analyzing associations within pairs. However, unlike in a randomized clinical trial, analyzing associations within family pairs cannot isolate unmeasured nonshared confounders.

An additional drawback of sibling comparisons is that they often generate less precise estimates because only pairs who are discordant for both the exposure and outcome are informative. For instance, despite the fact that hierarchical factor model scores in childhood have been found to be associated with several outcomes (eg, self-rated suicidal behavior, psychiatric diagnoses, and criminal convictions) in young adulthood (N = 8500 twin pairs), the associations became nonsignificant after adjusting for unmeasured confounds shared by twin pairs.^10,11^ Thus, larger sample sizes are needed when estimating associations with rare outcomes. The goal of this study was to use twin and sibling samples to examine whether associations between general and specific psychopathology factors and later outcomes remained after adjusting for unmeasured confounds shared by increasingly related family members.

## Methods

### Swedish Registers

This study used data from the Total Population Register, the Study of Twin Adults: Genes and Environment (STAGE), the National Patient Register, the Cause of Death Register, the Register of Convicted Offenders, the Register of Suspected Offenders, and the Prescribed Drug Register. All registers included follow-up until the end of 2013 except the Prescribed Drug Register, which included follow-up till the end of 2017. Ethical approval was granted by the regional ethical review board in Stockholm. Participant consent was waived for the sibling sample because deidentified data were used. Twin participants provided written participation consent. The study followed the Strengthening the Reporting of Observational Studies in Epidemiology (STROBE) reporting guideline, and data were analyzed from January 2022 to February 2023.

### Participants

A flowchart of the sample selection can be found in eFigure 1 in Supplement 1. In brief, from November 2004 to May 2006, all twins born in Sweden from 1959 to 1985 per the Swedish Twin Registry were invited to participate in STAGE (response rate = 60%), with the aim of assessing health in adult twins aged 20 to 45 years.^22^ We excluded twins who migrated or died (except for death from suicide) before the end of the study period. The final sample consisted of 10 432 dizygotic pairs and 5732 monozygotic pairs.

We included the oldest pair of siblings in each family born in Sweden from 1959 to 1985 per the Multi-Generation Register. After excluding those who died (except due to death from suicide) or migrated before the end of the study period, the sample consisted of 864 626 full sibling pairs and 106 427 maternal half-sibling pairs.

### Measures

#### Exposures Recorded Prior to 2005

In August 2005, twins participating in STAGE self-reported on 48 symptoms pertaining to mental health, all of which were included in this analysis (Table 1). The response formats were trichotomous (no; yes, to some extent; and yes) or binary (no and yes). To facilitate estimation of tetrachoric correlations, we combined the yes, to some extent responses with the yes responses.

**Table 1.** Exploratory Factor Loadings^a^ on 48 Self-reported Symptoms and Model Fit^b^ in the Twin Sample

Symptom	General factor loadings	*P* value	Specific internalizing factor loadings	*P* value	Specific neuro-develop-mental factor loadings	*P* value	Specific substance factor loadings	*P* value	Specific impulsivity factor loadings	*P* value
Have you ever felt sad, blue, or depressed for 2 or more weeks in a row?	0.51	<.001	0.45	<.001	0.13	<.001	0.21	<.001	−0.28	<.001
Have you ever had a period lasting one month or longer when most of the time you felt worried and anxious?	0.52	<.001	0.45	<.001	0.15	<.001	0.20	<.001	−0.28	<.001
Do you have a tendency toward excessive cleaning: hand washing, baths, showers, toothbrushing, etc?	0.39	<.001	0.61	<.001	−0.10	.81	−0.20	.59	0.09	.58
Do you take other special measures to avoid dirt, germs, or poisons?	0.37	<.001	0.60	<.001	−0.12	.78	−0.19	.61	0.08	.57
Do you have a tendency toward excessive checking: electric switches, gas taps, locks, doors, the oven, etc?	0.30	<.001	0.49	<.001	−0.07	.85	−0.18	.53	0.06	.66
Do you repeat the same simple activity many times in a row for no reason, such as repeatedly standing up or sitting down or going backward and forward through a doorway?	0.41	<.001	0.60	<.001	−0.18	.63	−0.12	.79	0.11	.39
Do you have a tendency toward touching things or people in particular ways?	0.41	<.001	0.58	<.001	−0.16	.66	−0.10	.81	0.09	.45
Do you have a tendency toward arranging things so they are just so, or exactly symmetrical?	0.40	<.001	0.52	<.001	−0.10	.80	−0.15	.68	0.13	.29
Do you have a tendency toward counting to particular lucky numbers or avoiding unlucky numbers?	0.33	<.001	0.49	<.001	−0.15	.63	−0.10	.79	0.08	.41
Do you have or have you ever had depression?	0.57	<.001	0.46	<.001	0.19	<.001	0.22	<.001	−0.30	<.001
Do you have or have you ever had panic attacks?	0.52	<.001	0.41	<.001	0.14	.003	0.20	<.001	−0.23	<.001
Do you have or have you ever had phobia?	0.37	<.001	0.35	<.001	0.10	.40	0.03	.68	−0.11	.17
Do you have difficulty expressing emotions and reactions with facial gestures, pronunciation, or body language?	0.20	.53	0.04	.91	0.49	<.001	−0.17	.65	−0.15	.71
Have you had difficulty getting and keeping friends?	0.29	.20	0.13	.61	0.38	<.001	−0.08	.78	−0.15	.62
Are you disinterested in sharing joy, interests, and activities with others?	0.20	.21	0.07	.67	0.25	.005	−0.08	.63	−0.04	.85
Can you only be with other people on your terms?	0.44	<.001	0.12	.25	0.26	.28	−0.02	.94	0.08	.69
Was your language development delayed?	0.15	.28	−0.05	.73	0.22	.09	−0.09	.63	0.07	.71
Do you have difficulty participating in discussions with others?	0.32	.34	0.11	.78	0.54	<.001	−0.15	.71	−0.18	.68
Do you have difficulty imitating other people or playing charades?	0.08	.74	0.00	.99	0.34	.005	−0.19	.48	−0.07	.84
Do you get absorbed in your interests to the extent of being repetitive or too intense?	0.40	<.001	0.08	.27	0.20	.50	−0.05	.83	0.18	.31
Do you get absorbed by routines in such a way as to produce problems for yourself or for others?	0.47	<.001	0.25	.02	0.20	.50	−0.11	.62	0.12	.57
Do you get absorbed by details?	0.45	<.001	0.17	.01	0.18	.54	−0.07	.77	0.16	.37
Thinking about your whole life, have you ever had motor tics involving any of the following types of repeated movement? Excessive blinking of the eyes, squinting your eyes, rolling your eyes upward, twitching your nose, flaring your nostrils, pouting your mouth, stretching your mouth wide open, nodding your head, screwing up your face, touching your chin or shoulder, stretching your neck, shrugging your shoulders, jerking movement of your arm or leg, or other motor tics?	0.22	<.001	0.17	.001	0.00	.97	0.06	.52	0.00	.89
Thinking about your whole life, have you ever had vocal tics involving any of the following types of repeated sounds? Excessive sniffing, coughing as a habit, gulping, high-pitched squeaks, making little noises (eg, ah, eh, eee), sucking noises, burping not just when eating or drinking, a word said repeatedly and out of context, swearing without meaning to and without being annoyed, or other vocal tics?	0.25	<.001	0.17	<.001	0.03	.85	−0.01	.95	0.06	.14
Do you often fail to pay close attention to details or make careless mistakes when you write or during other activities?	0.41	.003	−0.18	.07	0.32	.43	0.01	.99	0.26	.27
Do you often have difficulty sustaining attention in tasks or activities?	0.56	.005	−0.07	.67	0.44	.27	0.01	.99	0.18	.56
Do you often seem not to listen when spoken to directly?	0.49	<.001	−0.09	.22	0.30	.49	0.02	.96	0.27	.21
Do you often fail to follow instructions and to finish tasks?	0.56	.02	−0.03	.87	0.49	.17	−0.01	.99	0.12	.74
Do you often have difficulty organizing tasks and activities?	0.46	.12	0.00	.99	0.57	.001	−0.03	.94	−0.07	.86
Do you often avoid tasks that require sustained mental effort?	0.48	.04	0.03	.91	0.46	.05	−0.03	.94	0.02	.95
Do you often lose things?	0.42	.01	−0.20	.17	0.42	.25	0.10	.83	0.10	.70
Are you often easily distracted or disturbed?	0.54	.004	0.01	.96	0.41	.20	0.01	.97	0.10	.72
Are you often forgetful in daily activities?	0.46	.01	−0.17	.31	0.45	.21	0.10	.83	0.07	.80
Have you ever thought that you should limit your alcohol consumption?	0.43	.22	−0.02	.96	−0.18	.77	0.69	<.001	−0.05	.92
Have other people irritated you by criticizing your way of drinking?	0.46	.11	−0.02	.95	−0.10	.85	0.60	.001	−0.01	.97
Have you ever felt bad or have you ever had feelings of guilt due to your way of drinking?	0.47	.17	−0.01	.98	−0.15	.80	0.68	<.001	−0.05	.91
Have you ever been drinking the first thing in the morning to calm your nerves or to cure a hangover?	0.38	.04	0.00	.99	−0.02	.96	0.44	.001	−0.04	.89
Have you ever tried marijuana?	0.34	.31	−0.10	.79	−0.16	.78	0.64	<.001	−0.04	.93
Have you ever tried hash?	0.38	.23	−0.08	.84	−0.14	.81	0.64	<.001	−0.04	.93
Do you have difficulty holding your hands and feet still or staying seated?	0.46	<.001	−0.01	.81	0.15	.74	−0.07	.86	0.38	.02
Do you get up and move about in situations where you are supposed to remain seated?	0.50	<.001	0.01	.86	0.24	.55	0.00	.99	0.25	.17
Are you restless?	0.51	<.001	−0.01	.86	0.09	.86	−0.04	.94	0.46	<.001
Do you have difficulty engaging in calm leisure activities?	0.43	<.001	−0.03	.50	0.08	.88	−0.09	.83	0.47	<.001
Does it often feel like you are “on the go”?	0.47	<.001	−0.01	.95	−0.01	.99	−0.08	.87	0.57	<.001
Do you often talk excessively?	0.32	.001	−0.05	.80	−0.12	.80	−0.02	.96	0.51	<.001
Do you often blurt out answers before the question has been completed?	0.45	<.001	−0.07	.65	−0.03	.96	0.01	.99	0.54	<.001
Do you have difficulty waiting for your turn?	0.49	<.001	−0.06	.67	0.00	.99	0.02	.97	0.53	<.001
Do you often interrupt or intrude on others?	0.43	<.001	−0.06	.66	−0.02	.97	0.02	.97	0.50	<.001

^a^ Factors standardized to a mean of 0 and a variance of 1.

^b^ Root mean square error of approximation = 0.015; 90% CI = 0.015-0.015; confirmatory fit index = 0.922; Tucker-Lewis index = 0.917; χ^2^ = 19 138.333; *df* = 4494; *P* < .001.

For the siblings, we counted psychiatric diagnoses assigned prior to the mean of the STAGE assessment (August 4, 2005), such that the follow-up times were roughly equal for the twin and sibling samples. We linked the siblings to diagnoses of depression, anxiety, obsessive-compulsive disorder, posttraumatic stress disorder, bipolar disorder, schizophrenia, schizoaffective disorder, alcohol misuse, and drug use identified in the National Patient Register, which captures psychiatric inpatient admissions in Sweden since 1973 and outpatient admissions since 2001. For alcohol and drug use, we excluded diagnoses related to overdoses (which were included as an outcome), such that this exposure primarily captured symptoms related to withdrawal and dependence. The diagnoses were assigned by the attending physician using a nonhierarchical diagnostic structure in accordance with the *International Classification of Diseases*, *Eighth Revision (ICD-8)* (1969-1986), *Ninth Reivision* (*ICD-9*) (1987-1996), or *Tenth Revision* (*ICD-10*) (1997-present) (eTable 1 in Supplement 1 displays the *ICD* codes for each diagnosis). Diagnoses were recorded as lifetime events (up to August 4, 2005) and treated as binary variables.

#### Outcomes Recorded From 2005 to 2017

We linked the twin and sibling samples to 7 outcomes that occurred after the STAGE assessment (twin sample) or after the mean of the STAGE assessment (sibling sample). We combined diagnoses of suicide from the National Patient Register and death from suicide from the Cause of Death Register into an outcome. We combined diagnoses of overdoses of alcohol and drugs into an outcome. We combined conviction or suspicion of violent (eg, homicide, assault, and unlawful threats) and property crimes (eg, vehicle theft, burglary, and shoplifting) into an outcome. Using the Prescribed Drug Register, we combined prescription of antialcohol and antiopioid medications into 1 outcome; we combined prescription of antianxiety, sedatives, and selective serotonin reuptake inhibitors; and we combined prescription of antipsychotics, antiepileptics, and lithium. We also included prescription of stimulants (eTables 1 and 2 in Supplement 1 display the corresponding *ICD* and Anatomical Therapeutic Chemical codes, respectively). We treated all outcomes as binary categorical variables (ie, ever experienced the outcome during the follow-up period vs not) (eTable 3 in Supplement 1 displays outcome prevalence and pair discordance by pedigree). The follow-up time was 9 years for outcomes related to suicide, criminality, and overdoses (ie, until the end of 2013) and 13 years for the prescription outcomes (ie, until the end of 2017).

### Statistical Analyses

#### Hierarchical Factor Model Identification

We fit exploratory factor analyses to the 48 self-reported symptoms in the twin sample and to the 9 psychiatric diagnoses in the sibling sample, respectively. We extracted factors based on the scree plot, which pits the eigenvalues against the number of extracted dimensions.^23^ We rotated the factors to 1 general and several (unassociated) specific factors.^24^ We used the weighted least square mean and variance estimator in Mplus software version 8.3 (Muthén & Muthén).

#### Associations Between Hierarchical Factor Models and Outcomes

First, we regressed the outcomes onto the latent hierarchical factor models without adjusting for unmeasured family-constant confounding within an exploratory structural equation modeling framework.^25^ We excluded those who experienced the outcome prior to the exposure assessment to rule out reverse causation. We estimated odds ratios (ORs) because the outcomes were binary, with age and sex included as covariates.

**Figure 1.**
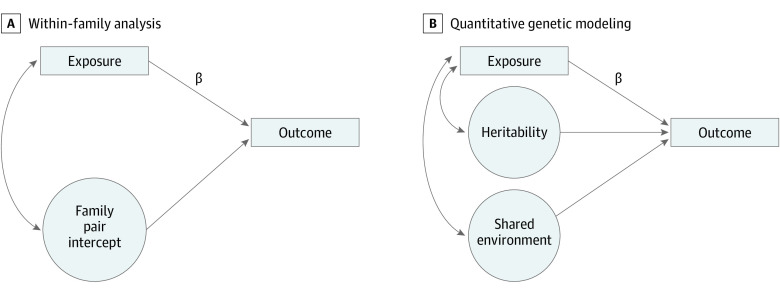
Association Between Exposure and Outcome After Adjusting for Unmeasured Confounds That Make Family Pairs Similar A, Allowing a latent family pair outcome intercept to correlate with the exposure adjusts the exposure-outcome association (β) for all unmeasured confounds that make pairs within a family similar. B, By applying quantitative genetic modeling to full and maternal half-siblings to estimate the heritability and shared environment components of the outcome and by allowing these to correlate with the exposure, one can mimic the monozygotic cotwin control design.

Second, we estimated odds ratios within pairs.^19,20,21^ This involved using a previously described approach^26^ to estimate a normally distributed latent outcome sibling pair intercept capturing everything that makes pairs within a family alike and allowing this pair intercept to correlate with the exposure (ie, the hierarchical factor model) (Figure 1A; eFigure 2 in Supplement 1).

Third, we applied quantitative genetic modeling to the full and maternal half-sibling samples to estimate the heritability and shared environment component of each outcome and allowed these to correlate with the exposure (Figure 1B; eFigure 3 in Supplement 1) to control for as much unmeasured confounding as the more stringent co-monozygotic control design in the sibling sample at the cost of making the traditional quantitative genetic modeling assumptions. We labeled this the AC model, as heritability is typically labeled A and shared environment C.^27^ We report 95% CIs corresponding to an α significance level of .05). We applied a false discovery rate correction for all associations (n = 217).

### Sensitivity Analysis

First, to examine whether the results depended on the latent variable framework (which assumes that the indicators are partly unreliable and that the latent factors are normally distributed), we computed individual scores on the first principal component (PC1), which makes no assumptions about unreliability or latent distributions, based on the Pearson correlations among the 48 symptoms and 9 disorders, respectively, using the psych package in R version 2.2.5 (R Foundation). We then regressed each outcome on PC1 using fixed-effects regression, which (unlike our original approach) makes no distributional assumptions about the pair intercepts.^28^

Second, to examine whether applying logistic regression to time-to-event data biased the results, we conducted Cox regression using the survival package in R version 5.3-0 (R Foundation) with PC1 as the exposure. Participants were censored at age at migration, death, or end of follow-up.

Third, to examine whether the results based on the STAGE survey might generalize to nonresponders, we identified opposite-sex sibling pairs born on the same day (ie, dizygotic twins) who did not respond to the STAGE survey. We then estimated associations between PC1 (based on 8 psychiatric diagnoses from which there was no missingness) and the outcomes separately for opposite-sex twins who responded vs did not respond to the STAGE survey.

## Results

### Hierarchical Factor Models

#### Twin Sample

For the twin sample (N = 32 328; mean [SD] age, 34 [8] years; 16 076 [49.73%] male), the first 10 eigenvalues for the 48 symptoms were 11.16, 4.00, 3.33, 3.09, 2.04, 1.71, 1.54, 1.26, 1.14, and 1.12. We extracted 4 factors based on the scree plot, and rotated the solution toward a hierarchical model consisting of 1 general and 4 specific factors (Table 1). The mean (range) standardized loading on the general factor equaled 0.41 (0.08-0.57). The first specific factor captured internalizing problems, the second neurodevelopmental problems, the third substance misuse, and the fourth problems related to impulsivity. As a sensitivity analysis, we also extracted a 6-factor solution (eTable 4 in Supplement 1).

#### Sibling Sample

For the sibling sample (N = 1 942 106; mean [SD] age, 34 [7] years; 991 500 [51.05%] male), the eigenvalues for the 9 disorders were 4.69, 1.24, 0.98, 0.67, 0.41, 0.36, 0.27, 0.24, and 0.14. We extracted 3 factors because that nearly satisfied the greater-than-unity recommendation^29^ and rotated the solution toward a hierarchical model (Table 2). The mean (range) standardized loading on the general factor equaled 0.60 (0.50-0.67). The first specific factor captured internalizing problems, the second substance problems, and the third psychotic problems.

**Table 2.** Exploratory Factor Loadings^a^ on 9 Psychiatric Diagnoses and Model Fit^b^ in the Sibling Sample

Diagnosis	General factor loadings	*P* value	Specific internalizing factor loadings	*P* value	Specific substance misuse factor loadings	*P* value	Specific psychotic factor loadings	*P* value
Depression	0.66	<.001	0.57	<.001	0.01	<.001	0.07	<.001
Anxiety	0.60	<.001	0.43	<.001	0.14	<.001	0.03	<.001
OCD	0.50	<.001	0.38	<.001	−0.04	.01	0.15	<.001
PTSD	0.50	<.001	0.49	<.001	0.09	<.001	−0.09	<.001
Alcohol	0.58	<.001	0.16	<.001	0.44	<.001	−0.02	.26
Drugs	0.67	<.001	0.04	.02	0.64	<.001	−0.01	.59
Bipolar	0.62	<.001	0.23	<.001	−0.08	.007	0.47	<.001
Schizophrenia	0.58	<.001	−0.04	.01	0.10	.001	0.51	<.001
Schizoaffective	0.65	<.001	−0.02	.31	−0.05	.36	0.73	<.001

Abbreviations: OCD, obsessive-compulsive disorder; PTSD, posttraumatic stress disorder.

^a^ Factors standardized to a mean of 0 and a variance of 1.

^b^ Root mean square error of approximation = 0.003; 90% CI = 0.003-0.003; confirmatory fit index = 0.995; Tucker-Lewis index = 0.995; χ^2^ = 1907.033; *df* = 399; *P* < .001.

### Associations Between Hierarchical Factor Models and Outcomes

#### Twin Sample

**Figure 2.**
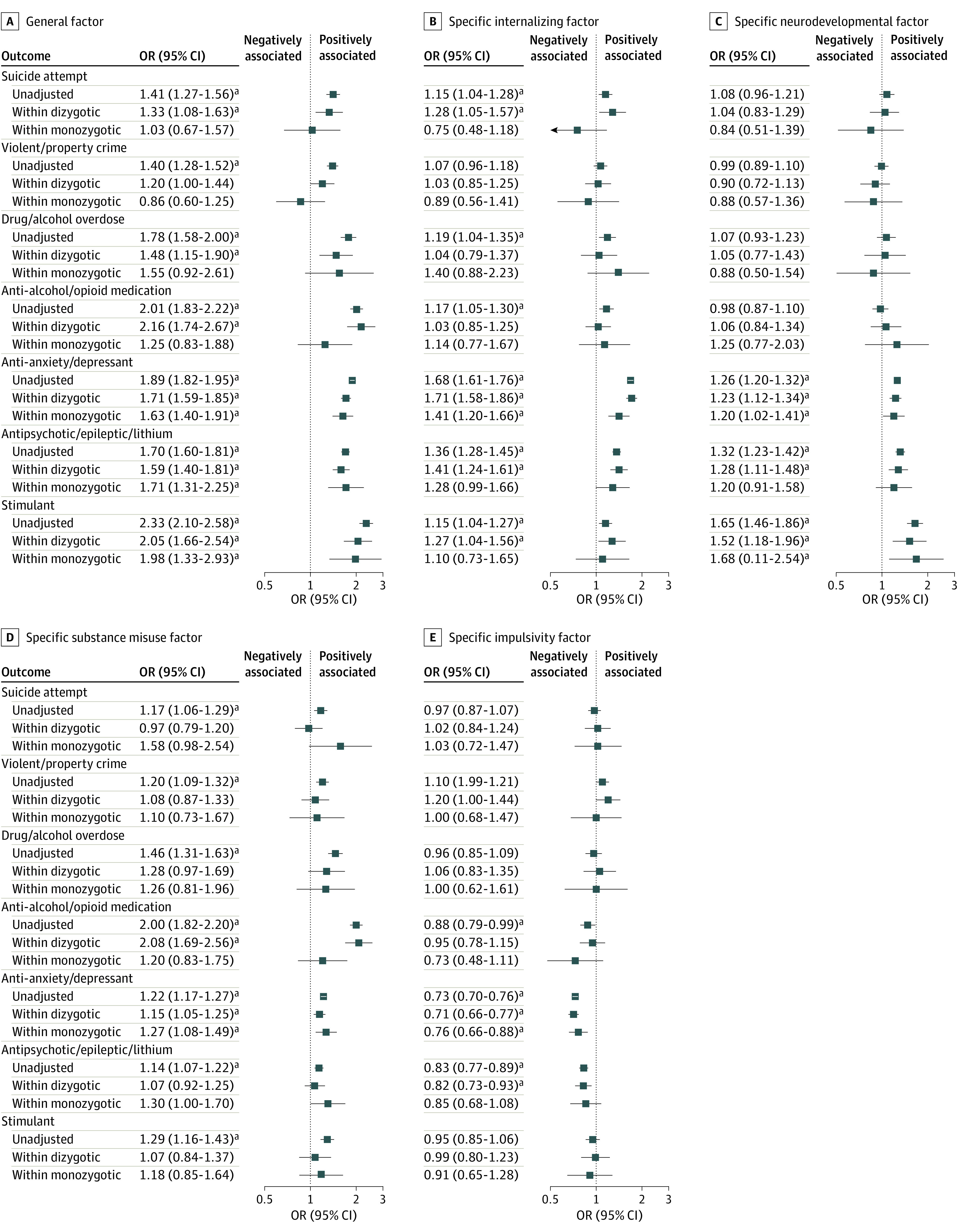
Associations Between General and Specific Factors and Later Outcomes in the Twin Sample Unadjusted indicates there was no adjusting for unmeasured confounds shared by twin pairs; within dizygotic and within monozygotic, after adjusting for unmeasured confounds shared by dizygotic and monozygotic twin pairs, respectively. eTable 5 in Supplement 1 includes estimates, CIs, and false discovery rate–corrected *P* values. ^a^Odds ratios met criteria for false discovery rate statistical significance.

Without adjusting for unmeasured confounds shared by twin pairs, as displayed in Figure 2 (eTable 5 in the Supplement includes estimates, CIs, and false discovery rate significance), the general factor was significantly associated with all 7 outcomes (mean OR, 1.79; 95% CI, 1.64-1.95). After adjusting for unmeasured confounds shared by dizygotic twin pairs, the general factor was significantly associated with all 7 outcomes (mean within-pairs OR, 1.65; 95% CI, 1.38-1.98). After adjusting for unmeasured confounds shared by monozygotic pairs, the general factor was significantly associated prescription of antidepressants, antipsychotics, and stimulants (mean within-pairs OR, 1.77; 95% CI, 1.35-2.36), but not the remaining 4 outcomes (mean within-pairs OR, 1.17; 95% CI, 0.75-1.83).

The specific internalizing factor was associated with prescription of antidepressants (OR, 1.68; 95% CI, 1.61-1.76), even after adjusting for shared monozygotic twin pair confounds (within-pairs OR, 1.41; 95% CI, 1.20-1.66). The specific neurodevelopmental factor was associated with prescription of stimulants (OR, 1.65; 95% CI, 1.46-1.86), even after adjusting for shared monozygotic twin pair confounds (within-pairs OR, 1.68; 1.11-2.54). The specific substance misuse factor was associated with prescription of antialcohol and antiopioid medication (OR, 2.00; 95% CI, 1.82-2.20); however, this association became nonsignificant after adjusting for confounds shared by monozygotic twin pairs (within-pairs OR, 1.20; 95% CI, 0.83-1.75). The associations remained similar for the 6-factor solution (eTable 6 in Supplement 1), suggesting that the results appeared robust to number of extracted factors.

#### Sibling Sample

**Figure 3.**
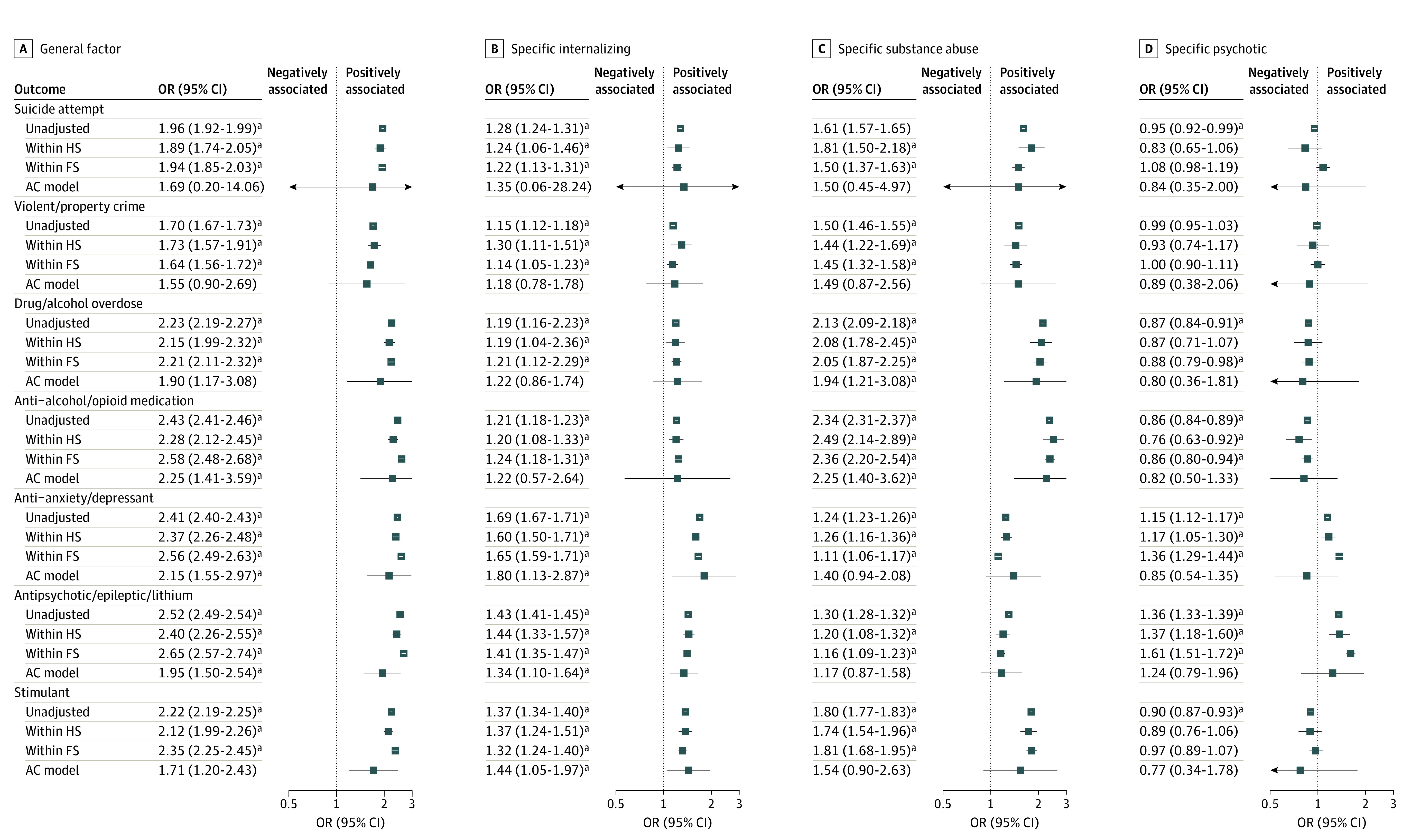
Associations Between General and Specific Factors and Later Outcomes in the Sibling Sample AC model indicates after adjusting for estimated 100% genetics and shared environment, where A refers to heritability and C refers to shared environment; unadjusted, no adjusting for unmeasured confounds shared by sibling pairs; within HS, after adjusting for unmeasured confounds shared by maternal half-sibling pairs; within FS, after adjusting for unmeasured confounds shared by full sibling pairs. eTable 7 in Supplement 1 includes estimates, CIs, and false discovery rate–corrected *P* values. ^a^Odds ratios met criteria for false discovery rate statistical significance.

Without adjusting for unmeasured confounds shared by sibling pairs, as displayed in Figure 3 (eTable 7 in Supplement 1 includes all β values, CIs, and false discovery rate significance), the general factor was significantly associated with all 7 outcomes (mean OR, 2.21; 95% CI, 2.18-2.24). All general factor-outcome associations remained significant after adjusting for confounds shared by maternal half sibling pairs (mean within-pairs OR, 2.13; 95% CI, 1.99-2.29) and full sibling pairs (mean within-pairs OR = 2.28; 95% CI, 2.19-2.37). In the AC model, the general factor was significantly associated with 5 outcomes (mean AC OR, 1.99; 95% CI, 1.97-2.92) but not suicidal (AC OR, 1.69; 95% CI, 0.20-14.06) or criminal behavior (OR, 1.55; 0.90-2.69).

The specific internalizing factor was associated with prescription of antidepressants (OR, 1.69; 95% CI, 1.67-1.71), even after adjusting for unmeasured confounds shared by full siblings (within-pairs OR, 1.65; 95% CI, 1.59-1.71). The specific substance misuse factor was associated with prescription of antialcohol and antiopioid medication (OR, 2.34; 95% CI, 2.31-2.37), even after adjusting for unmeasured confounds shared by full siblings (within-pairs OR, 2.36; 95% CI, 2.20-2.54). The specific psychotic factor was most strongly associated with prescription of antipsychotic medications (OR, 1.36; 95% CI, 1.33-1.39), even after adjusting for unmeasured confounds shared by full siblings (within-pairs OR, 1.61; 95% CI, 1.51-1.72).

### Sensitivity Analysis Results

eTable 8 in Supplement 1 displays the PC1 loadings for the twin sample. eTable 9 and eFigure 4 in Supplement 1 show that within dizygotic twin pairs, PC1 was significantly associated with 6 outcomes (mean within-pairs OR, 1.34; 95% CI, 1.16-1.54) but not criminality (within-pair OR, 1.04; 95% CI, 0.92-1.17). Within monozygotic pairs, PC1 was significantly associated with the same 3 outcomes (mean within-pairs OR, 1.37; 95% CI, 1.15-1.67) as the latent general factor. eTable 10 in Supplement 1 displays the PC1 loadings for the sibling sample. eTable 11 and eFigure 5 in Supplement 1 show that PC1 was significantly associated with all outcomes within maternal half sibling pairs (mean within-pairs OR_, _1.45; 95% CI, 1.40-1.51) and full sibling pairs (mean within-pairs OR, 1.47; 95% CI, 1.44-1.50). eTables 9 and 11 in Supplement 1 show that the hazard and odds ratios between PC1 and the outcomes estimates were similar.

eTable 12 in Supplement 1 displays the PC1 loadings on psychiatric diagnoses for the opposite-sex twin sample. eTable 13 in Supplement 1 shows that the associations between PC1 and the outcomes were similar among opposite-sex twins who responded to vs those who did not respond to the STAGE survey, except that the associations with suicide and antidepressant prescription were weaker among nonresponders within pairs.

## Discussion

Factors on a latent hierarchical factor model based on self-reported symptoms and psychiatric diagnoses, respectively, remained associated with clinically relevant outcomes within twin and sibling pairs. This suggests that the associations likely cannot be attributed to unmeasured confounds shared by family members. One possibility is that the associations might be attributable to unmeasured confounds not shared by family members, such as partners or friends. Alternatively, the factors might be in the causal pathway toward the outcomes. If so, interventions geared toward broad psychopathology dimensions might reduce the probability of future clinically relevant events.

### Implications

It might be useful to examine the effect of treatment not only on specific psychiatric diagnoses, but also on broad dimensions of psychopathology. For instance, psychologists have recently integrated different anxiety treatments into a single module to treat the internalizing spectrum.^30,31^ While speculative, this study also raises the possibility that an even broader set of treatment principles might help reduce a general liability toward all mental health problems. Two observational studies^32,33^ found that general psychopathology decreased substantially following therapy. Likewise, in a randomized clinical trial^34^ of 136 institutionalized 2-year-old children in Romania, those randomized to high-quality foster care were rated 0.3 standard deviations lower on general psychopathology in adolescence compared to those randomized to remain in institutions. Together, these results raise the possibility that general psychopathology might change following interventions.

### Lack of Attenuation With Outcomes Within Families

Interestingly, the hierarchical factor-outcome associations did not attenuate within pairs. Whereas previous studies of children might simply have been underpowered to detect within-pair associations,^10,11^ another possibility is that the rank order stability within family members (ie, whether the same sibling scores higher at both time 1 and 2) is relatively low in childhood but increases in adulthood.^35^ Small initial mental health differences between siblings in childhood might snowball into larger differences in adulthood due to environmental selection and reinforcement, such that adjusting for shared family unmeasured confounds attenuates associations less in adulthood than childhood.^36^

### Limitations

The results of this study should be interpreted in light of limitations. First, importantly, the co-twin control and sibling comparison designs cannot isolate unmeasured confounds not shared by family members. Randomized clinical trials remain the gold standard for inferring causality because they rule out all potential confounds.

Second, because co-twin control and sibling comparison designs only analyze pairs that have different scores (ie, those that are discordant), they can amplify confounding not shared by pair members.^37^ This might be more likely to occur if only a small subset of the pairs are discordant. However, most pairs were discordant for the outcomes in our samples, suggesting that our within-pair analyses were not restricted to small subsamples. More generally, this amplification occurs if the exposures (ie, the factors) are more correlated between pair members than the unmeasured confounders. Thus, the within-pair estimates in this study might be overestimated if the associations are accounted for by confounders, such as partner or peer effects, which are likely less correlated than the exposures. Alternatively, the within-pair estimates are likely relatively unbiased if the associations are accounted for by confounders, such as family background variables or genetics, which are likely more correlated than the exposures.

Third, the response rate in the twin sample was relatively low. Nevertheless, the associations between PC1 based on nonmissing psychiatric diagnoses and the outcomes were quite similar for responders and nonresponders, suggesting that the relatively low response rate might not have exerted a strong bias on the results. Furthermore, the sibling sample, which had no missing data, generated similar results. Fourth, although general psychopathology might be a malleable construct, it remains uncertain if it is possible to develop a set of treatment principles for it. Fifth, although our twin and sibling samples were large, it remains unknown whether the results generalize outside of Sweden.

## Conclusion

In this study, across 2 adult samples and 2 assessment modalities, the associations between general and specific psychopathology factors and clinically relevant outcomes tended to remain after isolating unmeasured confounds shared by family members. These results indicate that treating broader psychopathology dimensions might reduce the probability of future clinically relevant events.

## Notes

Supplement 1. eTable 1. International Classification of Diseases (ICD) codes for psychiatric diagnoses. eTable 2. Anatomical Therapeutic Chemical (ATC) codes. eTable 3. Prevalence of outcomes occurring after 2005. eTable 4. Six-factor hierarchical factor loadings on 48 self-reported symptoms in the twin sample. eTable 5. Associations between the hierarchical factor model and outcomes in the twin sample. eTable 6. Associations between six-factor hierarchical model and outcomes in the twin sample. eTable 7. Associations between general factor model and outcomes in the sibling sample. eTable 8. Standardized loadings on the first principal component in the twin sample. eTable 9. Associations between first principal component (PC1) and outcomes in the twin sample estimated via logistic regression (left) and Cox regression (right). eTable 10. Standardized loadings on the first principal component in the sibling sample. eTable 11. Associations between first principal component (PC1) and outcomes in the sibling sample estimated via logistic regression (left) and Cox regression (right). eTable 12. Standardized loadings on the first principal component (PC1) of psychiatric diagnoses in the opposite-sex twin sample. eTable 13. Associations between first principal component (PC1) of psychiatric diagnoses and outcomes among opposite-sex dizygotic (DZO) twin STAGE survey responders and non-responders. eFigure 1. Flow chart of the twin and sibling sample selection. eFigure 2. Explanation of the within-pair model. eFigure 3. Explanation of the AC-model. eFigure 4. Fixed effects associations between first principal component and later outcomes in twin sample. eFigure 5. Fixed effects associations between first principal component and later outcomes in sibling sample.

Supplement 2. Data sharing statement
