# Supplementary material for: Associations Between General and Specific Psychopathology Factors and 10-Year Clinically Relevant Outcomes in Adult Swedish Twins and Siblings
Source: JAMA Psychiatry. 2023 May 10;80(7):728–37. doi: 10.1001/jamapsychiatry.2023.1162 (PMC10173102; doi:10.1001/jamapsychiatry.2023.1162)
Supplement: Supplement 1. — eTable 1. International Classification of Diseases (ICD) codes for psychiatric diagnoses. eTable 2. Anatomical Therapeutic Chemical (ATC) codes. eTable 3. Prevalence of outcomes occurring after 2005. eTable 4. Six-factor hierarchical factor loadings on 48 self-reported symptoms in the twin sample. eTable 5. Associations between the hierarchical factor model and outcomes in the twin sample. eTable 6. Associations between six-factor hierarchical model and outcomes in the twin sample. eTable 7. Associations between general factor model and outcomes in the sibling sample. eTable 8. Standardized loadings on the first principal component in the twin sample. eTable 9. Associations between first principal component (PC1) and outcomes in the twin sample estimated via logistic regression (left) and Cox regression (right). eTable 10. Standardized loadings on the first principal component in the sibling sample. eTable 11. Associations between first principal component (PC1) and outcomes in the sibling sample estimated via logistic regression (left) and Cox regression (right). eTable 12. Standardized loadings on the first principal component (PC1) of psychiatric diagnoses in the opposite-sex twin sample. eTable 13. Associations between first principal component (PC1) of psychiatric diagnoses and outcomes among opposite-sex dizygotic (DZO) twin STAGE survey responders and non-responders. eFigure 1. Flow chart of the twin and sibling sample selection. eFigure 2. Explanation of the within-pair model. eFigure 3. Explanation of the AC-model. eFigure 4. Fixed effects associations between first principal component and later outcomes in twin sample. eFigure 5. Fixed effects associations between first principal component and later outcomes in sibling sample. [file jamapsychiatry-e231162-s001.pdf]

Supplemental Online Content

Pettersson E, Larsson H, D'Onofrio BM, Lichtenstein P. Associations between general and specific psychopathology factors and 10-year clinically relevant outcomes in adult Swedish twins and siblings. *JAMA Psychiatry*. Published online May 10, 2023. doi:10.1001/jamapsychiatry.2023.1162

eTable 1. International Classification of Diseases (ICD) codes for psychiatric diagnoses.

eTable 2. Anatomical Therapeutic Chemical (ATC) codes.

eTable 3. Prevalence of outcomes occurring after 2005.

eTable 4. Six-factor hierarchical factor loadings on 48 self-reported symptoms in the twin sample.

eTable 5. Associations between the hierarchical factor model and outcomes in the twin sample.

eTable 6. Associations between six-factor hierarchical model and outcomes in the twin sample.

eTable 7. Associations between general factor model and outcomes in the sibling sample.

eTable 8. Standardized loadings on the first principal component in the twin sample.

eTable 9. Associations between first principal component (PC1) and outcomes in the twin sample estimated via logistic regression (left) and Cox regression (right).

eTable 10. Standardized loadings on the first principal component in the sibling sample.

eTable 11. Associations between first principal component (PC1) and outcomes in the sibling sample estimated via logistic regression (left) and Cox regression (right).

eTable 12. Standardized loadings on the first principal component (PC1) of psychiatric diagnoses in the opposite-sex twin sample.

eTable 13. Associations between first principal component (PC1) of psychiatric diagnoses and outcomes among opposite-sex dizygotic (DZO) twin STAGE survey responders and non-responders.

eFigure 1. Flow chart of the twin and sibling sample selection.

© 2023 Pettersson E et al. *JAMA Psychiatry.*

eFigure 2. Explanation of the within-pair model.

eFigure 3. Explanation of the AC-model.

eFigure 4. Fixed effects associations between first principal component and later outcomes in twin sample.

eFigure 5. Fixed effects associations between first principal component and later outcomes in sibling sample.

This supplemental material has been provided by the authors to give readers additional information about their work.

eTable 1. International Classification of Diseases (ICD) codes for psychiatric diagnoses.

Disorder	ICD-8	ICD-9	ICD-10
Depression	3004	300E, 311	F32-F39
Anxiety	300 (excl. 3003, 3004)	300 (excl. 300D, 300E)	F40-41, F44-45, F48
Obsessive-compulsive disorder	3003	300D	F42
Post-traumatic stress disorder	307	308, 309	F43
Bipolar disorder	2960, 2963, 2968, 2969	296A-E, 296W, 296X	F30-31
Schizophrenia	2950-4, 2956, 2958-9	295A-E, 295G, 295W, 295X	F20
Schizoaffective disorder	2957	295H	F25
Alcohol use	291, 303	291, 303, 305A	F10 (excl. F105)
Drug abuse	304	292, 304, 305.X (excl. 305A)	F11-F19 (excl. F1X.5)
Suicide attempts	-	-	X60-X84, Y10-Y34
Substance overdose	-	-	F100-F190

© 2023 Pettersson E et al. *JAMA Psychiatry.*

eTable 2. Anatomical Therapeutic Chemical (ATC) codes.

Drug	ATC
Anxiolytics, sedatives, antidepressants	
Benzodiazepine	N05BA
Diphenylmethane	N05BB
Carbamates	N05BC
Dibenzo-bicyclo-octadiene	N05BD
Azaspirodecanedione	N05BE
Other anxiolytics	N05BX
Barbiturates, plain	N05CA
Barbiturates, combinations	N05CB
Aldehydes	N05CC
Benzodiazepine	N05CD
Piperidinedione	N05CE
Benzodiazepine related drugs	N05CF
Melatonin receptor agonists	N05CH
Other hypnotics and sedatives	N05CM
Hypnotics and sedatives in combination, excluding barbiturates	N05CX
Non-selective monoamine reuptake inhibitors	N06AA
Selective serotonin reuptake inhibitors	N06AB
Monoamine oxidase inhibitors, non-selective	N06AF
Monoamine oxidase A inhibitors	N06AG
Other antidepressants	N06AX
Stimulants	
Centrally acting sympathomimetics	N06BA
Xanthine derivatives	N06BC
Other psychostimulants and nootropics	N06BX
Antipsychotics, lithium, and antiepileptics	
Phenothiazines with aliphatic side-chain	N05AA
Phenothiazines with piperazine structure	N05AB
Phenothiazines with piperidine structure	N05AC
Butyrophenone derivatives	N05AD
Indole derivatives	N05AE
Thioxanthene derivative	N05AF
Diphenylbutylpiperidine derivatives	N05AG
Diazepines, oxazepines, thiazepines and oxepines	N05AH
Neuroleptics, in tardive dyskinesia	QN05AK
Benzamides	N05AL
Other antipsychotics	N05AX
Lithium	N05AN
Barbiturates	N03AA
Hydantoin	N03AB
Oxazolidine	N03AC
Succinimide	N03AD
Benzodiazepine	N03AE
Carboxamide	N03AF

Fatty acid	N03AG
Other antiepileptics	N03AX
Drugs against alcohol or opioid dependence	
Disulfiram	N07BB01
Calcium carbimide	N07BB02
Acamprosate	N07BB03
Naltrexone	N07BB04
Nalmefene	N07BB05
Buprenorphine	N07BC01
Methadone	N07BC02
Levacetylmethadol	N07BC03
Lofexidine	N07BC04
Levomethadone	N07BC05
Diamorphine	N07BC06
Buprenorphine, combinations	N07BC51

© 2023 Pettersson E et al. *JAMA Psychiatry.*

eTable 3. Prevalence of outcomes occurring after 2005.

Outcome	DZ twin sample (no/yes/discordant pairs)	MZ twin sample (no/yes/discordant pairs)	Maternal half-sibling sample (no/yes/discordant pairs)	Full sibling sample (no/yes/discordant pairs)
Suicide attempt	20,023/ 253/ 230	11,058/ 139/ 115	189,603/ 3,667/ 3,549	1,622,579/ 18,625/ 17,989
Violent/ property crime	18,360/ 417/ 332	10,175/ 223/ 137	131,921/ 5,105/ 4,817	1,347,713/ 29,583/ 28,041
Drug/alcohol overdose	20,529/ 179/ 173	11,255/ 114/ 98	203,040/ 3,312/ 3,214	1,689,626/ 13,526/ 13,162
Anti-alcohol/ opioid medication	20,549/ 305/ 279	11,324/ 138/ 102	206,510/ 5,878/ 5,650	1,700,997/ 26,351/ 24,963
Anti-anxiety/ depressant	15,600/ 4,783/ 3,195	8.563/ 2,655/ 1,393	137,168/ 63,520/ 41,008	1,256,884/ 398,778/ 273,952
Antipsychotic/ epileptic/ lithium	19,868/ 906/ 800	10,959/ 472/ 374	196,958/ 14,052/ 12,712	1,640,359/ 77,167/ 70,785
Stimulant	20,636/ 220/	11,354/ 110/	207,110/ 5,488/	1,706,214/ 21,808/

© 2023 Pettersson E et al. *JAMA Psychiatry.*

	208	82	5,162	20,484

Note. Participants who experienced the outcome prior to the STAGE assessment (twins) or the mean of the STAGE assessment (siblings) are excluded.

© 2023 Pettersson E et al. *JAMA Psychiatry.*

Table 4. Six-factor hierarchical factor loadings on 48 self-reported symptoms in the twin sample.

Symptom	General factor	Specific internalizing factor	Specific substance misuse factor	Specific OCD factor	Specific impulsivity factor	Specific autism factor	Specific inattention factor
Have you ever felt sad, blue or depressed for two weeks or more in a row?	**0.51**	**0.64**	0.02	0.03	-0.08	-0.06	-0.04
Have you ever had a period lasting one month or longer when most of the time you felt worried and anxious?	**0.51**	**0.64**	0.02	0.04	-0.09	-0.06	-0.04
Excessive cleaning: hand washing, baths, showers, toothbrushing etc?	**0.44**	0.08	-0.12	**0.63**	-0.03	0.00	-0.12
Other special measures to avoid dirt, germs or poisons?	**0.42**	0.06	-0.10	**0.62**	-0.04	-0.01	-0.12
Excessive checking: electric switches, gas taps, locks, doors, the oven?	**0.35**	0.02	-0.10	**0.52**	-0.04	0.04	-0.09
Repeating the same simple activity many times	**0.44**	0.04	-0.01	**0.64**	-0.04	-0.08	-0.11

© 2023 Pettersson E et al. *JAMA Psychiatry.*

in a row for no reason, e.g. repeatedly standing up or sitting down or going backwards and forwards through a doorway?							
Touching things or people in particular ways?	**0.44**	0.03	0.01	**0.62**	-0.06	-0.07	-0.09
Arranging things so they are just so, or exactly symmetrical?	**0.45**	-0.02	-0.04	**0.58**	0.01	0.02	-0.09
Counting to particular lucky numbers or avoiding unlucky numbers?	**0.36**	0.00	0.00	**0.56**	-0.06	-0.07	-0.08
Do you have or have you ever had depression?	**0.58**	**0.65**	0.03	0.01	-0.09	0.00	-0.03
Do you have or have you ever had panic attacks?	**0.54**	**0.56**	0.06	0.04	-0.06	-0.02	-0.04
Do you have or have you ever had phobia?	**0.41**	0.28	-0.02	0.19	-0.08	0.05	-0.02
Do you have difficulties expressing emotions and reactions	**0.32**	-0.10	-0.11	-0.01	-0.17	**0.51**	0.20

© 2023 Pettersson E et al. *JAMA Psychiatry.*

with facial gestures, pronunciation, or body language?							
Have you difficulties to get and keep friends?	**0.37**	0.09	-0.08	0.03	-0.14	**0.30**	0.18
Are you disinterested in sharing joy, interests, and activities with others?	0.24	0.04	-0.09	0.01	-0.03	0.19	0.12
Can you only be with other people on your terms?	**0.48**	0.01	0.00	0.09	0.07	0.15	0.16
Were your language development delayed?	0.18	-0.08	-0.08	-0.03	0.07	0.15	0.16
Do you have difficulties participating in discussions with others?	**0.44**	0.00	-0.11	0.00	-0.19	**0.50**	0.23
Do you have difficulty imitating other people or to play charades?	0.17	-0.11	-0.15	-0.03	-0.07	**0.40**	0.12
Do you get absorbed by your interests in such a way as being repetitive or too intense?	**0.42**	-0.01	-0.05	0.08	0.17	0.07	0.15
Do you get absorbed by routines in such a way	**0.52**	0.04	-0.08	0.21	0.12	0.17	0.07

© 2023 Pettersson E et al. *JAMA Psychiatry.*

as to produce problems for yourself or for others?							
Do you get absorbed by details?	**0.48**	0.01	-0.05	0.17	0.14	0.08	0.13
Thinking about the whole of your life, have you ever had motor tics involving any of the following types of repeated movement? Excessive blinking of eyes	0.23	0.06	0.07	0.17	-0.05	-0.07	0.05
Thinking about the whole of your life, have you ever had vocal tics involving any of the following types of repeated sounds?	0.27	-0.01	0.02	0.20	0.01	0.00	0.04
Do you often fail to pay close attention to details or make careless mistakes when you write, or other activities?	**0.40**	-0.13	0.00	-0.09	0.22	0.01	**0.39**

Do you often have difficulty sustaining attention in tasks or activities?	**0.59**	-0.05	0.00	-0.07	0.17	0.14	**0.40**
Do you often seem not to listen when spoken to directly?	**0.49**	-0.12	0.03	0.01	0.22	0.00	**0.36**
Do you often fail to follow instructions and to finish tasks?	**0.60**	-0.05	-0.01	-0.03	0.08	0.19	**0.42**
Do you often have difficulty organising tasks and activities?	**0.52**	0.03	-0.06	-0.06	-0.10	0.28	**0.43**
Do you often avoid tasks that require sustained mental effort?	**0.53**	0.03	-0.04	-0.02	0.00	0.21	**0.35**
Do you often lose things?	**0.37**	0.05	-0.03	-0.10	0.03	-0.18	**0.60**
Are you often easily distracted or disturbed?	**0.55**	0.08	-0.05	-0.01	0.08	0.04	**0.42**
Are you often forgetful in daily activities?	**0.42**	0.07	-0.03	-0.09	-0.01	-0.14	**0.61**
Have you ever thought that you should limit your alcohol consumption ?	**0.44**	-0.07	**0.73**	-0.05	-0.05	0.06	-0.19
Have other people irritated you	**0.47**	-0.08	**0.63**	-0.04	-0.02	0.09	-0.12

© 2023 Pettersson E et al. *JAMA Psychiatry.*

by criticizing your way of drinking?							
Have you ever felt bad or have you had feelings of guilt due to your way of drinking?	**0.48**	-0.07	**0.72**	-0.03	-0.05	0.09	-0.17
Have you ever been drinking the first thing in the morning to calm your nerves or to cure a hangover	**0.40**	0.03	**0.47**	-0.07	-0.03	0.06	-0.06
Have you ever tried Marijuana	0.25	0.14	**0.59**	-0.11	-0.08	**-0.43**	0.13
Have you ever tried hash	**0.30**	0.16	**0.59**	-0.10	-0.08	**-0.41**	0.13
Do you have difficulties holding your hands and feet still or can you not stay seated?	**0.46**	0.01	-0.07	-0.08	**0.45**	0.10	0.06
Do you get up and move about in situations when you are supposed to remain seated?	**0.51**	0.04	-0.01	-0.07	**0.30**	0.10	0.15
Are you restless?	**0.50**	0.06	-0.04	-0.16	**0.57**	0.11	-0.04
Do you have difficulty doing calm leisure pursuit?	**0.42**	-0.02	-0.07	-0.13	**0.56**	0.13	-0.05
Does it often feel like you	**0.44**	0.01	-0.07	-0.09	**0.66**	0.03	-0.10

© 2023 Pettersson E et al. *JAMA Psychiatry.*

are ”on the go”?							
Do you often talk excessively?	0.24	-0.06	-0.04	0.07	**0.52**	**-0.31**	0.07
Do you often blurt out answers before the question has been completed?	**0.38**	-0.15	0.01	0.11	**0.50**	-0.29	0.20
Do you have difficulty awaiting turns?	**0.43**	-0.13	0.02	0.09	**0.50**	-0.24	0.19
Do you often interrupt or intrude on others?	**0.36**	-0.15	0.02	0.13	**0.44**	-0.30	0.21

Note. Loadings greater than .29 are bolded for visual clarity.
Root mean square error of approximation [RMSEA] = 0.010, 90% confidence interval [CI] = 0.010, 0.011; Confirmatory fit index [CFI] = 0.963; Tucker-Lewis index [TLI]= 0.960; χ^2 = 11266.491, degrees of freedom [df] = 4383, $p <$ 0.001.

© 2023 Pettersson E et al. *JAMA Psychiatry.*

eTable 5. Associations between the hierarchical factor model and outcomes in the twin sample.

	Exposure				
Outcome	General factor	Specific internalizing factor	Specific neurodevelop-mental factor	Specific substance misuse factor	Specific impulsivity factor
Suicide	1.41* (1.27, 1.56)	1.15* (1.04, 1.28)	1.08 (0.96, 1.21)	1.17* (1.06, 1.29)	0.97 (0.89, 1.07)
Suicide within DZ pairs	1.33* (1.08, 1.63)	1.28* (1.05, 1.57)	1.04 (0.83, 1.29)	0.97 (0.79, 1.20)	1.02 (0.84, 1.24)
Suicide within MZ pairs	1.03 (0.67, 1.57)	0.75 (.48, 1.18)	0.84 (0.51, 1.39)	1.58 (0.98, 2.54)	1.03 (0.72, 1.47)
Crime	1.40* (1.28, 1.52)	1.07 (0.96, 1.18)	0.99 (0.89, 1.10)	1.20* (1.09, 1.32)	1.10 (0.99, 1.21)
Crime within DZ pairs	1.20 (1.00, 1.44)	1.03 (0.85, 1.25)	0.90 (0.72, 1.13)	1.08 (0.87, 1.33)	1.20 (1.00, 1.44)
Crime within MZ pairs	0.86 (0.60, 1.25)	0.89 (0.56, 1.41)	0.88 (0.57, 1.36)	1.10 (0.73, 1.67)	1.00 (0.68, 1.47)
Substance overdose	1.78* (1.58, 2.00)	1.19* (1.04, 1.35)	1.07 (0.93, 1.23)	1.46* (1.31, 1.63)	0.96 (0.85, 1.09)
Substance overdose within DZ pairs	1.48* (1.15, 1.90)	1.04* (0.79, 1.37)	1.05 (0.77, 1.43)	1.28 (0.97, 1.69)	1.06 (0.83, 1.35)
Substance overdose within MZ pairs	1.55 (0.92, 2.60)	1.40* (0.88, 2.23)	0.88 (0.50, 1.54)	1.26 (0.81, 1.96)	1.00 (0.62, 1.61)
Substance misuse medication	2.01* (1.83, 2.22)	1.17* (1.05, 1.30)	0.98 (0.87, 1.10)	2.00* (1.82, 2.20)	0.88* (0.79, 0.99)
Substance misuse medication within DZ pairs	2.16* (1.74, 2.67)	1.03 (0.85, 1.25)	1.06 (0.84, 1.34)	2.08* (1.69, 2.56)	0.94 (0.78, 1.15)
Substance misuse medication within MZ pairs	1.25 (0.83, 1.88)	1.14 (0.77, 1.67)	1.25 (0.77, 2.03)	1.20 (0.83, 1.75)	0.73 (0.48, 1.11)
Antidepressants	1.89* (1.82, 1.95)	1.68* (1.61, 1.76)	1.26* (1.20, 1.32)	1.22* (1.17, 1.27)	0.73* (0.70, 0.76)
Antidepressants within DZ pairs	1.71* (1.59, 1.85)	1.71* (1.58, 1.86)	1.23* (1.12, 1.34)	1.15* (1.05, 1.25)	0.71* (0.66, 0.77)

© 2023 Pettersson E et al. *JAMA Psychiatry.*

Antidepressants within MZ pairs	1.63* (1.40, 1.91)	1.41* (1.20, 1.66)	1.20* (1.02, 1.41)	1.27* (1.08, 1.49)	0.76* (0.66, 0.88)
Antipsychotics	1.70* (1.60, 1.81)	1.36* (1.28, 1.45)	1.32* (1.23, 1.42)	1.14* (1.07, 1.22)	0.82* (0.77, 0.89)
Antipsychotics within DZ pairs	1.59* (1.40, 1.81)	1.41* (1.24, 1.61)	1.28* (1.11, 1.48)	1.07 (0.92, 1.24)	0.82* (0.73, 0.93)
Antipsychotics within MZ pairs	1.71* (1.31, 2.25)	1.28 (0.99, 1.66)	1.20 (0.91, 1.58)	1.30 (1.00, 1.70)	0.85 (0.67, 1.08)
Stimulants	2.33* (2.10, 2.58)	1.15* (1.05, 1.27)	1.65* (1.46, 1.86)	1.29* (1.16, 1.43)	0.95 (0.85, 1.06)
Stimulants within DZ pairs	2.05* (1.66, 2.54)	1.27* (1.04, 1.56)	1.52* (1.18, 1.96)	1.07 (0.84, 1.37)	0.99 (0.80, 1.23)
Stimulants within MZ pairs	1.98* (1.33, 2.93)	1.10 (0.73, 1.65)	1.68* (1.11, 2.54)	1.17 (0.85, 1.64)	0.90 (0.65, 1.28)

Note. Estimates represent odds ratios (95% confidence intervals). MZ = monozygotic. DZ = dizygotic. * = significant at $p < 0.05$ after False Discovery Rate correction (n = 217 tests).

© 2023 Pettersson E et al. *JAMA Psychiatry.*

eTable 6. Associations between six-factor hierarchical model and outcomes in the twin sample.

	Exposure						
Outcome	General factor	Specific internalizing factor	Specific substance misuse factor	Specific OCD factor	Specific impulsivity factor	Specific autism factor	Specific inattention factor
Suicide	1.42 (1.27, 1.58)	1.31 (1.18, 1.46)	1.10 (0.99, 1.23)	0.93 (0.84, 1.03)	1.07 (0.96, 1.20)	1.01 (0.89, 1.14)	0.97 (0.87, 1.10)
Suicide within DZ pairs	1.36 (1.09, 1.69)	1.36 (1.09, 1.69)	0.98 (0.78, 1.24)	1.20 (0.97, 1.49)	0.99 (0.79, 1.23)	0.94 (0.74, 1.19)	1.05 (0.84, 1.31)
Suicide within MZ pairs	0.90 (0.56, 1.46)	1.14 (0.78, 1.68)	1.14 (0.78, 1.68)	0.55 (0.35, 0.87)	1.32 (0.83, 2.11)	0.99 (0.55, 1.78)	0.80 (0.52, 1.22)
Crime	1.39 (1.26, 1.52)	1.11 (1.01, 1.23)	1.20 (1.08, 1.33)	0.95 (0.86, 1.06)	1.14 (1.02, 1.27)	0.93 (0.83, 1.03)	0.99 (0.88, 1.12)
Crime within DZ pairs	1.22 (1.01, 1.48)	0.77 (0.64, 0.93)	1.21 (0.96, 1.53)	1.10 (0.91, 1.34)	1.12 (0.90, 1.40)	1.10 (0.89, 1.36)	0.88 (0.71, 1.11)
Crime within MZ pairs	0.85 (0.54, 1.34)	0.90 (0.63, 1.29)	1.22 (0.74, 2.02)	0.92 (0.54, 1.56)	0.93 (0.56, 1.55)	0.81 (0.47, 1.40)	1.03 (0.69, 1.53)
Substance overdose	1.81 (1.60, 2.04)	1.39 (1.23, 1.57)	1.40 (1.24, 1.59)	0.84 (0.75, 0.94)	1.09 (0.95, 1.26)	1.04 (0.91, 1.18)	0.91 (0.78, 1.08)
Substance overdose within DZ pairs	1.87 (1.57, 2.22)	1.37 (1.16, 1.63)	1.24 (1.05, 1.47)	0.90 (0.76, 1.07)	1.12 (0.94, 1.33)	1.09 (0.93, 1.28)	0.97 (0.78, 1.20)
Substance overdose within MZ pairs	1.57 (0.88, 2.78)	1.57 (1.04, 2.36)	1.25 (0.73, 2.16)	1.06 (0.64, 1.77)	1.15 (0.65, 2.04)	0.67 (0.37, 1.19)	0.90 (0.55, 1.48)
Substance misuse medication	2.09 (1.88, 2.32)	1.36 (1.21, 1.51)	1.96 (1.77, 2.17)	0.72 (0.64, 0.80)	1.01 (0.89, 1.14)	1.15 (1.03, 1.29)	0.81 (0.71, 0.92)
Substance misuse medication within DZ pairs	2.22 (1.77, 2.77)	1.33 (1.10, 1.60)	1.94 (1.55, 2.43)	0.65 (0.53, 0.81)	1.17 (0.95, 1.44)	1.24 (0.97, 1.57)	0.82 (0.66, 1.03)

© 2023 Pettersson E et al. *JAMA Psychiatry.*

Substance misuse medication within MZ pairs	1.36 (0.83, 2.21)	1.38 (0.93, 2.06)	1.17 (0.73, 1.85)	0.90 (0.59, 1.38)	0.73 (0.44, 1.19)	1.06 (0.62, 1.81)	1.14 (0.76, 1.69)
Antidepressants	1.91 (1.83, 2.00)	2.11 (2.02, 2.21)	1.03 (0.97, 1.08)	1.00 (0.95, 1.06)	0.87 (0.82, 0.92)	1.03 (0.97, 1.09)	0.99 (0.94, 1.05)
Antidepressants within DZ pairs	1.73 (1.60, 1.87)	2.30 (2.12, 2.50)	0.90 (0.81, 0.99)	1.05 (0.96, 1.15)	0.92 (0.84, 1.01)	0.92 (0.83, 1.02)	0.99 (0.91, 1.08)
Antidepressants within MZ pairs	1.77 (1.47, 2.14)	1.47 (1.29, 1.67)	1.20 (0.98, 1.48)	1.00 (0.84, 1.19)	0.80 (0.66, 0.97)	1.17 (0.94, 1.45)	0.99 (0.85, 1.15)
Antipsychotics	1.76 (1.65, 1.88)	1.68 (1.57, 1.79)	0.99 (0.92, 1.06)	0.97 (0.90, 1.04)	0.98 (0.91, 1.05)	1.13 (1.05, 1.21)	1.03 (0.96, 1.11)
Antipsychotics within DZ pairs	1.66 (1.45, 1.90)	1.67 (1.49, 1.88)	0.92 (0.78, 1.09)	1.06 (0.92, 1.23)	0.93 (0.81, 1.07)	1.02 (0.88, 1.18)	1.09 (0.95, 1.26)
Antipsychotics within MZ pairs	1.73 (1.26, 2.36)	1.53 (1.24, 1.91)	1.19 (0.86, 1.65)	0.92 (0.70, 1.21)	1.02 (0.74, 1.42)	0.94 (0.66, 1.34)	1.04 (0.82, 1.33)
Stimulants	2.37 (2.12, 2.65)	1.65 (1.49, 1.82)	1.10 (0.98, 1.23)	0.82 (0.73, 0.91)	1.14 (1.01, 1.27)	1.09 (0.97, 1.24)	1.34 (1.19, 1.52)
Stimulants within DZ pairs	2.20 (1.76, 2.75)	1.43 (1.17, 1.75)	0.96 (0.73, 1.24)	0.99 (0.79, 1.25)	1.14 (0.89, 1.46)	1.25 (0.97, 1.60)	1.20 (0.95, 1.51)
Stimulants within MZ pairs	2.10 (1.33, 3.30)	1.37 (0.98, 1.93)	1.03 (0.68, 1.55)	0.82 (0.54, 1.23)	1.00 (0.63, 1.58)	1.47 (0.85, 2.53)	1.31 (0.92, 1.87)

Note. Estimates represent odds ratios (95% confidence intervals). MZ = monozygotic. DZ = dizygotic.

© 2023 Pettersson E et al. *JAMA Psychiatry.*

eTable 7. Associations between general factor model and outcomes in the sibling sample.

	Exposure			
Outcome	General factor	Specific internalizing factor	Specific substance misuse factor	Specific psychotic factor
Suicide	1.96* (1.92, 1.99)	1.28* (1.24, 1.31)	1.61* (1.57, 1.65)	0.95* (0.92, 0.99)
Suicide within maternal half-sibling pairs	1.87* (1.74, 2.05)	1.24* (1.06, 1.46)	1.81* (1.50, 2.18)	0.83 (0.65, 1.06)
Suicide within full sibling pairs	1.94* (1.85, 2.03)	1.22* (1.13, 1.31)	1.50* (1.37, 1.63)	1.08 (0.98, 1.19)
Suicide controlling 100% genetics and shared environment	1.69 (0.20, 14.06)	1.35 (0.06, 28.24)	1.50 (0.45, 4.94)	0.84 (0.35, 2.00)
Crime	1.70* (1.67, 1.73)	1.15* (1.12, 1.18)	1.50* (1.46, 1.55)	0.98 (0.95, 1.03)
Crime within maternal half-sibling pairs	1.73* (1.57, 1.91)	1.30* (1.11, 1.51)	1.44* (1.22, 1.69)	0.93 (0.73, 1.17)
Crime within full sibling pairs	1.64* (1.56, 1.72)	1.14* (1.05, 1.23)	1.45* (1.32, 1.58)	1.00 (0.90, 1.11)
Crime controlling 100% genetics and shared environment	1.55 (0.90, 2.69)	1.18 (0.78, 1.78)	1.49 (0.87, 2.56)	0.89 (0.38, 2.06)
Substance overdose	2.23* (2.19, 2.27)	1.19* (1.16, 1.23)	2.13* (2.09, 2.18)	0.87* (0.84, 0.91)
Substance overdose within maternal half-sibling pairs	2.15* (1.99, 2.32)	1.19* (1.04, 1.36)	2.08* (1.78, 2.45)	0.87 (0.71, 1.07)
Substance overdose within full sibling pairs	2.21* (2.11, 2.32)	1.21* (1.12, 1.29)	2.05* (1.87, 2.25)	0.88* (0.79, 0.98)
Substance overdose controlling 100% genetics	1.90* (1.17, 3.07)	1.22 (0.86, 1.74)	1.94* (1.21, 3.08)	0.80 (0.36, 1.81)

© 2023 Pettersson E et al. *JAMA Psychiatry.*

and shared environment				
Substance misuse medication	2.43* (2.41, 2.46)	1.21* (1.18, 1.23)	2.34* (2.31, 2.37)	0.86* (0.84, 0.89)
Substance misuse medication within maternal half-sibling pairs	2.28* (2.12, 2.45)	1.20* (1.08, 1.33)	2.49* (2.14, 2.89)	0.76* (0.63, 0.92)
Substance misuse medication within full sibling pairs	2.58* (2.48, 2.68)	1.24* (1.18, 1.31)	2.36* (2.20, 2.54)	0.87* (0.80, 0.94)
Substance misuse medication controlling 100% genetics and shared environment	2.25* (1.41, 3.59)	1.22 (0.57, 2.64)	2.25* (1.40, 3.62)	0.82 (0.50, 1.33)
Antidepressants	2.41* (2.40, 2.43)	1.69* (1.67, 1.71)	1.24* (1.23, 1.26)	1.15* (1.12, 1.17)
Antidepressants within maternal half-sibling pairs	2.37* (2.26, 2.48)	1.60* (1.50, 1.71)	1.26* (1.16, 1.36)	1.17* (1.05, 1.30)
Antidepressants within full sibling pairs	2.56* (2.49, 2.63)	1.65* (1.59, 1.71)	1.11* (1.06, 1.17)	1.36* (1.29, 1.44)
Antidepressants controlling 100% genetics and shared environment	2.15* (1.55, 2.97)	1.80* (1.13, 2.87)	1.40 (0.94, 2.08)	0.85 (0.54, 1.36)
Antipsychotics	2.52* (2.49, 2.54)	1.43* (1.41, 1.45)	1.30* (1.28, 1.32)	1.36* (1.33, 1.39)
Antipsychotics within maternal half-sibling pairs	2.39* (2.26, 2.55)	1.44* (1.33, 1.57)	1.20* (1.08, 1.32)	1.37* (1.18, 1.60)
Antipsychotics within full sibling pairs	2.65* (2.57, 2.74)	1.40* (1.35, 1.47)	1.16* (1.09, 1.23)	1.61* (1.51, 1.72)

© 2023 Pettersson E et al. *JAMA Psychiatry.*

Antipsychotics controlling 100% genetics and shared environment	1.95* (1.50, 2.54)	1.34* (1.10, 1.64)	1.17 (0.87, 1.58)	1.24 (0.78, 1.96)
Stimulants	2.22* (2.19, 2.25)	1.37* (1.34, 1.40)	1.80* (1.77, 1.83)	0.90* (0.87, 0.92)
Stimulants within maternal half-sibling pairs	2.12* (1.99, 2.26)	1.37* (1.24, 1.50)	1.74* (1.54, 1.96)	0.89 (0.76, 1.06)
Stimulants within full sibling pairs	2.35* (2.25, 2.45)	1.32* (1.24, 1.40)	1.81* (1.68, 1.95)	0.97 (0.89, 1.07)
Stimulants controlling 100% genetics and shared environment	1.71* (1.20, 2.43)	1.44* (1.05, 1.97)	1.54 (0.90, 2.63)	0.77 (0.34, 1.78)

Note. Estimates represent odds ratios (95% confidence intervals). MZ = monozygotic. DZ = dizygotic. * = significant at $p < 0.05$ after False Discovery Rate correction (n = 217 tests).

© 2023 Pettersson E et al. *JAMA Psychiatry.*

**eTable 8.** Standardized loadings on the first principal component in the twin sample.	
Symptom	First principal component
Have you ever felt sad, blue or depressed for two weeks or more in a row?	**0.38**
Have you ever had a period lasting one month or longer when most of the time you felt worried and anxious?	**0.39**
Excessive cleaning: hand washing, baths, showers, toothbrushing etc?	0.29
Other special measures to avoid dirt, germs or poisons?	0.27
Excessive checking: electric switches, gas taps, locks, doors, the oven?	0.27
Repeating the same simple activity many times in a row for no reason, e.g. repeatedly standing up or sitting down or going backwards and forwards through a doorway?	0.26
Touching things or people in particular ways?	0.28
Arranging things so they are just so, or exactly symmetrical?	**0.34**
Counting to particular lucky numbers or avoiding unlucky numbers?	0.25
Do you have or have you ever had depression?	**0.44**
Do you have or have you ever had panic attacks?	**0.38**
Do you have or have you ever had phobia?	0.28
Do you have difficulties expressing emotions and reactions with facial gestures, pronunciation, or body language?	0.25
Have you difficulties to get and keep friends?	**0.31**
Are you disinterested in sharing joy, interests, and activities with others?	0.16
Can you only be with other people on your terms?	**0.36**
Were your language development delayed?	0.15
Do you have difficulties participating in discussions with others?	**0.32**
Do you have difficulty imitating other people or to play charades?	0.19
Do you get absorbed by your interests in such a way as being repetitive or too intense?	**0.40**
Do you get absorbed by routines in such a way as to produce problems for yourself or for others?	**0.39**
Do you get absorbed by details?	**0.49**
Thinking about the whole of your life, have you ever had motor tics involving any of the following types of repeated movement? Excessive blinking of eyes	0.11
Thinking about the whole of your life, have you ever had vocal tics involving any of the following types of repeated sounds?	0.14
Do you often fail to pay close attention to details or make careless mistakes when you write, or other activities?	**0.44**
Do you often have difficulty sustaining attention in tasks or activities?	**0.57**
Do you often seem not to listen when spoken to directly?	**0.52**
Do you often fail to follow instructions and to finish tasks?	**0.50**
Do you often have difficulty organising tasks and activities?	**0.45**
Do you often avoid tasks that require sustained mental effort?	**0.46**
Do you often lose things?	**0.43**
Are you often easily distracted or disturbed?	**0.58**
Are you often forgetful in daily activities?	**0.48**
Have you ever thought that you should limit your alcohol consumption?	0.25
Have other people irritated you by criticizing your way of drinking?	0.25

© 2023 Pettersson E et al. *JAMA Psychiatry.*

Have you ever felt bad or have you had feelings of guilt due to your way of drinking?	0.28
Have you ever been drinking the first thing in the morning to calm your nerves or to cure a hangover	0.18
Have you ever tried Marijuana	0.17
Have you ever tried hash	0.20
Do you have difficulties holding your hands and feet still or can you not stay seated?	**0.47**
Do you get up and move about in situations when you are supposed to remain seated?	**0.41**
Are you restless?	**0.53**
Do you have difficulty doing calm leisure pursuit?	**0.40**
Does it often feel like you are ”on the go”?	**0.45**
Do you often talk excessively?	**0.31**
Do you often blurt out answers before the question has been completed?	**0.45**
Do you have difficulty awaiting turns?	**0.47**
Do you often interrupt or intrude on others?	**0.40**
Note. Loadings greater than .29 are bolded for visual clarity.	

© 2023 Pettersson E et al. *JAMA Psychiatry.*

eTable 9. Associations between first principal component (PC1) and outcomes in the twin sample estimated via logistic regression (left) and Cox regression (right).

	PC1 (odds ratios)	PC1 (hazard ratios)
Suicide	1.23 (1.16, 1.31)	1.21 (1.15, 1.28)
Suicide within DZ pairs	1.19 (1.03, 1.39)	1.20 (1.03, 1.39)
Suicide within MZ pairs	1.01 (0.81, 1.26)	1.00 (0.81, 1.23)
Crime	1.20 (1.15, 1.25)	1.21 (1.15, 1.28)
Crime within DZ pairs	1.04 (0.92, 1.18)	1.09 (0.95, 1.24)
Crime within MZ pairs	0.93 (0.75, 1.16)	0.98 (0.80, 1.19)
Substance overdose	1.30 (1.24, 1.37)	1.29 (1.23, 1.35)
Substance overdose within DZ pairs	1.19 (1.01, 1.40)	1.17 (1.00, 1.37)
Substance overdose within MZ pairs	1.20 (0.96, 1.49)	1.11 (0.90, 1.35)
Substance misuse medication	1.34 (1.28, 1.39)	1.30 (1.25, 1.34)
Substance misuse medication within DZ pairs	1.37 (1.13, 1.66)	1.35 (1.16, 1.56)
Substance misuse medication within MZ pairs	1.14 (0.91, 1.44)	1.12 (0.93, 1.35)
Antidepressants	1.41 (1.38, 1.44)	1.29 (1.27, 1.31)
Antidepressants within DZ pairs	1.36 (1.29, 1.43)	1.27 (1.22, 1.32)
Antidepressants within MZ pairs	1.22 (1.14, 1.32)	1.15 (1.09, 1.22)
Antipsychotics	1.35 (1.31, 1.39)	1.29 (1.26, 1.32)
Antipsychotics within DZ pairs	1.28 (1.17, 1.39)	1.25 (1.15, 1.35)
Antipsychotics within MZ pairs	1.17 (1.05, 1.32)	1.09 (0.99, 1.19)
Stimulants	1.50	1.44

© 2023 Pettersson E et al. *JAMA Psychiatry.*

	(1.43, 1.57)	(1.39, 1.50)
Stimulants within DZ pairs	1.57<br>(1.28, 1.92)	1.61<br>(1.33, 1.96)
Stimulants within MZ pairs	1.69<br>(1.24, 2.31)	1.29<br>(1.08, 1.55)
Note. Estimates represent odds (left) and hazard (right) ratios (95% confidence intervals). MZ = monozygotic. DZ = dizygotic.		

© 2023 Pettersson E et al. *JAMA Psychiatry.*

eTable 10. Standardized loadings on the first principal component in the sibling sample.

Diagnosis	First principal component
Depression	**0.60**
Anxiety	**0.55**
OCD	0.25
PTSD	**0.41**
Alcohol	**0.46**
Drugs	**0.47**
Bipolar	**0.55**
Schizophrenia	**0.36**
Schizoaffective	**0.37**

Note. Factor loadings greater than 0.29 are bolded for visual clarity.

© 2023 Pettersson E et al. *JAMA Psychiatry.*

**eTable 11.** Associations between first principal component (PC1) and outcomes in the sibling sample estimated via logistic regression (left) and Cox regression (right).		
	PC1 (odds ratios)	PC1 (hazard ratios)
Suicide	1.38 (1.37, 1.39)	1.34 (1.33, 1.35)
Suicide within maternal half-sibling pairs	1.42 (1.35, 1.49)	1.55 (1.40, 1.72)
Suicide within full sibling pairs	1.42 (1.39, 1.47)	1.41 (1.37, 1.45)
Crime	1.28 (1.27, 1.29)	1.34 (1.33, 1.35)
Crime within maternal half-sibling pairs	1.32 (1.26, 1.39)	1.31 (1.21, 1.40)
Crime within full sibling pairs	1.29 (1.27, 1.32)	1.27 (1.24, 1.30)
Substance overdose	1.43 (1.42, 1.44)	1.37 (1.36, 1.37)
Substance overdose within maternal half-sibling pairs	1.47 (1.50, 1.66)	1.57 (1.44, 1.71)
Substance overdose within full sibling pairs	1.51 (1.47, 1.55)	1.53 (1.48, 1.57)
Substance misuse medication	1.46 (1.46, 1.47)	1.35 (1.35, 1.36)
Substance misuse medication within maternal half-sibling pairs	1.48 (1.42, 1.53)	1.46 (1.39, 1.53)
Substance misuse medication within MZ pairs	1.53 (1.50, 1.56)	1.46 (1.43, 1.49)
Antidepressants	1.67 (1.66, 1.67)	1.30 (1.30, 1.31)
Antidepressants within maternal half-sibling pairs	1.49 (1.46, 1.52)	1.32 (1.29, 1.36)
Antidepressants within full sibling pairs	1.56 (1.54, 1.58)	1.35 (1.34, 1.37)
Antipsychotics	1.49 (1.49, 1.50)	1.34 (1.33, 1.34)
Antipsychotics within maternal half-sibling pairs	1.41 (1.38, 1.45)	1.31 (1.28, 1.35)
Antipsychotics within full sibling pairs	1.46	1.41

© 2023 Pettersson E et al. *JAMA Psychiatry.*

	(1.45, 1.48)	(1.39, 1.43)
Stimulants	1.41<br>(1.40, 1.41)	1.34<br>(1.34, 1.35)
Stimulants within maternal half-sibling pairs	1.48<br>(1.43, 1.54)	1.41<br>(1.34, 1.47)
Stimulants within full sibling pairs	1.50<br>(1.47, 1.54)	1.45<br>(1.42, 1.48)
Note. Estimates represent odds (left) and hazard (right) ratios (95% confidence intervals).		

eTable 12. Standardized loadings on the first principal component (PC1) of psychiatric diagnoses in the opposite-sex twin sample.

Diagnosis	PC1
Depression	**0.64**
Anxiety	**0.59**
OCD	0.26
PTSD	**0.40**
Alcohol	**0.53**
Drugs	**0.42**
Bipolar	**0.46**
Schizophrenia	**0.37**

Note. Factor loadings greater than 0.29 are bolded for visual clarity. Loadings based on Pearson correlations. Includes all opposite-sex twins contacted to participate in the STAGE study.

© 2023 Pettersson E et al. *JAMA Psychiatry.*

eTable 13. Associations between first principal component (PC1) of psychiatric diagnoses and outcomes among opposite-sex dizygotic (DZO) twin STAGE survey responders and non-responders.

Outcome	PC1 among DZO STAGE responders	PC1 among DZO STAGE non-responders
Suicide	1.55 (1.38, 1.74)	1.42 (1.31, 1.55)
Suicide within pairs	1.89 (1.14, 3.13)	1.06 (0.87, 1.30)
Crime	1.28 (1.13, 1.45)	1.36 (1.24, 1.49)
Crime within pairs	1.29 (0.99, 1.68)	1.23 (0.90, 1.67)
Substance overdose	1.50 (1.35, 1.66)	1.47 (1.36, 1.58)
Substance overdose within pairs	1.35 (1.01, 1.82)	1.44 (1.01, 2.04
Substance misuse medication	1.58 (1.47, 1.70)	1.43 (1.34, 1.53)
Substance misuse medication within pairs	1.57 (1.02, 2.42)	1.33 (1.05, 1.67)
Antidepressants	1.93 (1.74, 2.15)	1.62 (1.50, 1.76)
Antidepressants within pairs	1.74 (1.42, 2.13)	1.32 (1.14, 1.54)
Antipsychotics	1.45 (1.34, 1.58)	1.47 (1.38, 1.56)
Antipsychotics within pairs	1.40 (1.10, 1.77)	1.53 (1.19, 1.98)
Stimulants	1.44 (1.32, 1.57)	1.41 (1.30, 1.51)
Stimulants within pairs	1.21 (0.87, 1.68)	1.23 (0.93, 1.63)

Note. Estimates represent odds ratios (95% confidence intervals). PC1 scores are computed from loadings in eTable 10.

© 2023 Pettersson E et al. *JAMA Psychiatry.*

eFigure 1. Flow chart of the twin and sibling sample selection.

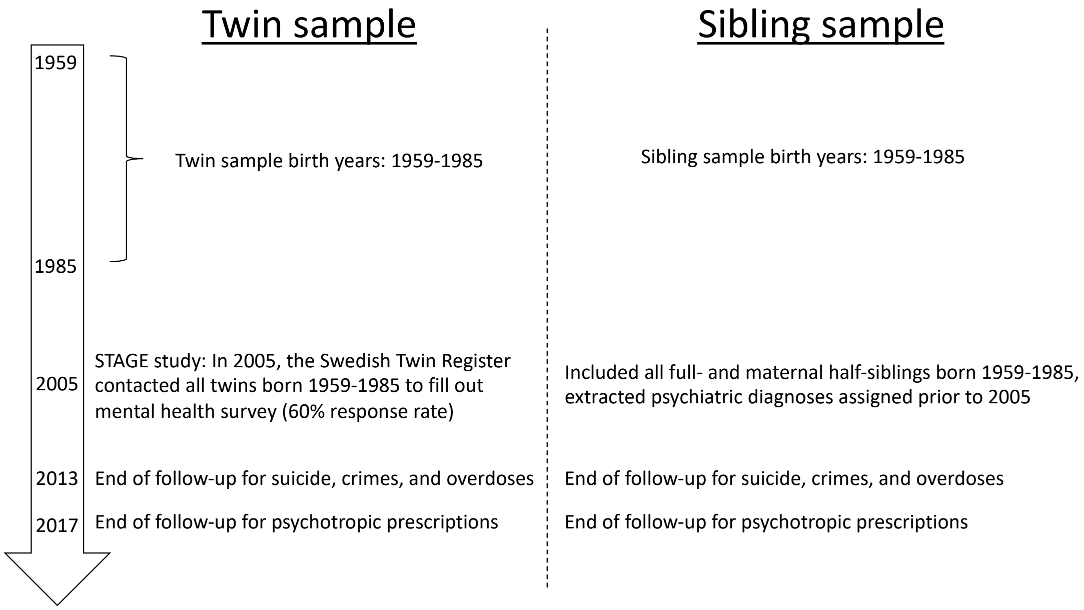

© 2023 Pettersson E et al. *JAMA Psychiatry.*

eFigure 2. Explanation of the within-pair model.

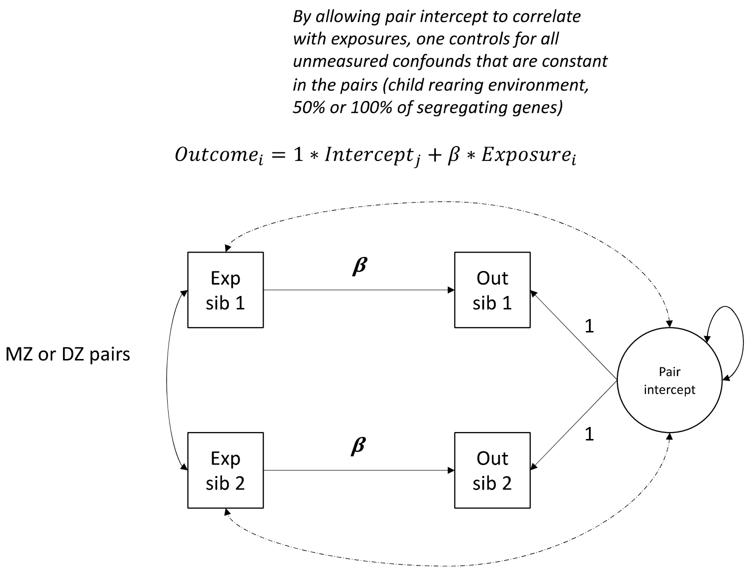

© 2023 Pettersson E et al. *JAMA Psychiatry.*

eFigure 3. Explanation of the AC-model.

By estimating genetic (A) and shared environment (C) via twin parameterization,
and allowing these to correlate with exposure,
we control for 100% of segregating genes and all environmental variables shared by sibling pairs

$$Outcome_i = a * A_j + c * C_j + \beta * Exposure_i$$

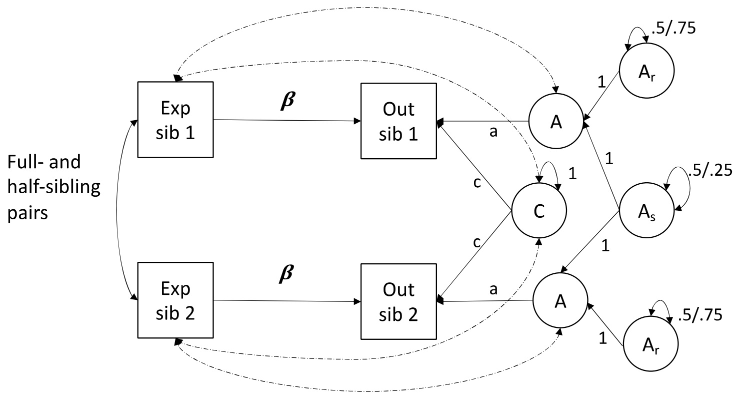

© 2023 Pettersson E et al. *JAMA Psychiatry.*

eFigure 4. Fixed effects associations between first principal component and later outcomes in twin sample.

Figure legend: Associations between first principal component of 48 symptoms and outcomes estimated using fixed effects regression. Unadjusted = without adjusting for unmeasured confounds shared by twin pairs. Within DZ = after adjusting for unmeasured confounds shared by dizygotic twin pairs. Within MZ = after adjusting for unmeasured confounds shared by monozygotic twin pairs.

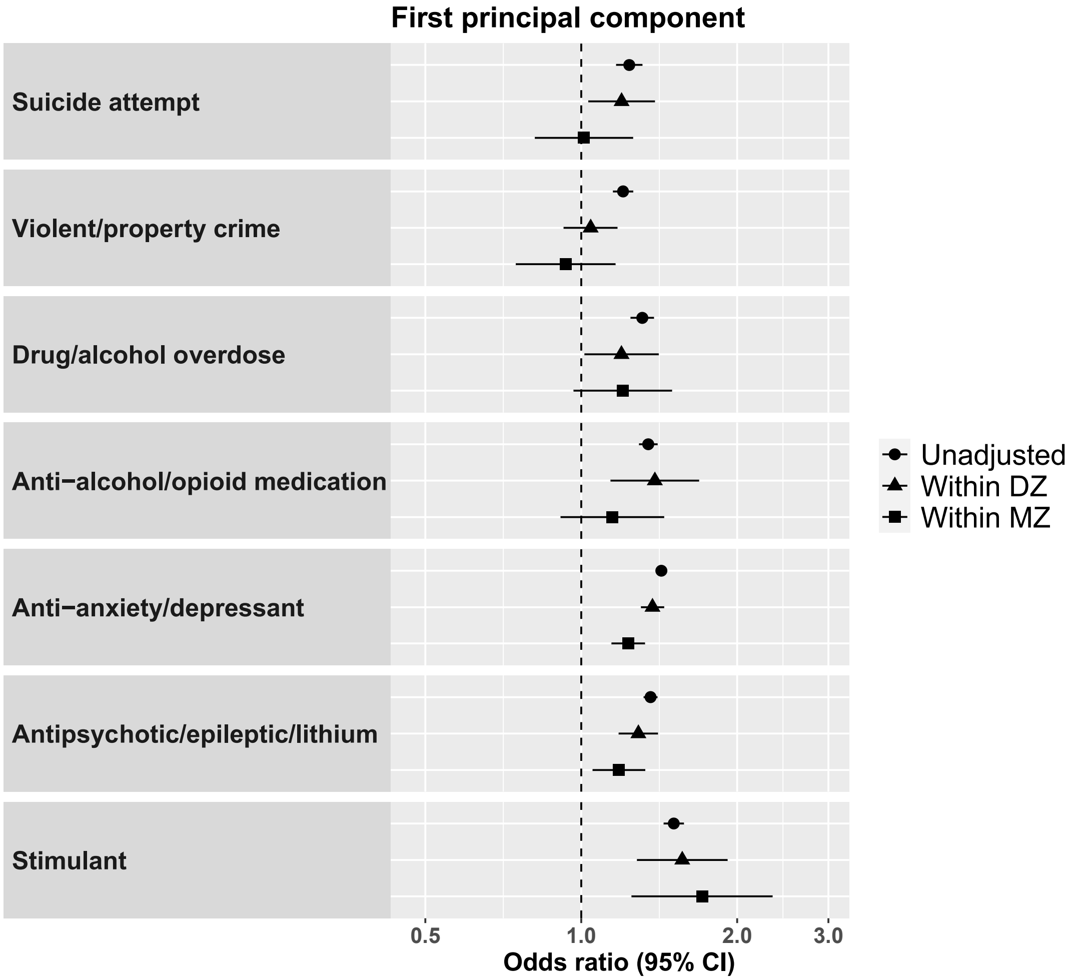

© 2023 Pettersson E et al. *JAMA Psychiatry.*

eFigure 5. Fixed effects associations between first principal component and later outcomes in sibling sample.

Figure legend: Associations between first principal component of 9 psychiatric disorders and outcomes estimated using fixed effects regression. Unadjusted = without adjusting for unmeasured confounds shared by sibling pairs. Within HS = after adjusting for unmeasured confounds shared by maternal half-sibling pairs. Within FS = after adjusting for unmeasured confounds shared by full sibling pairs.

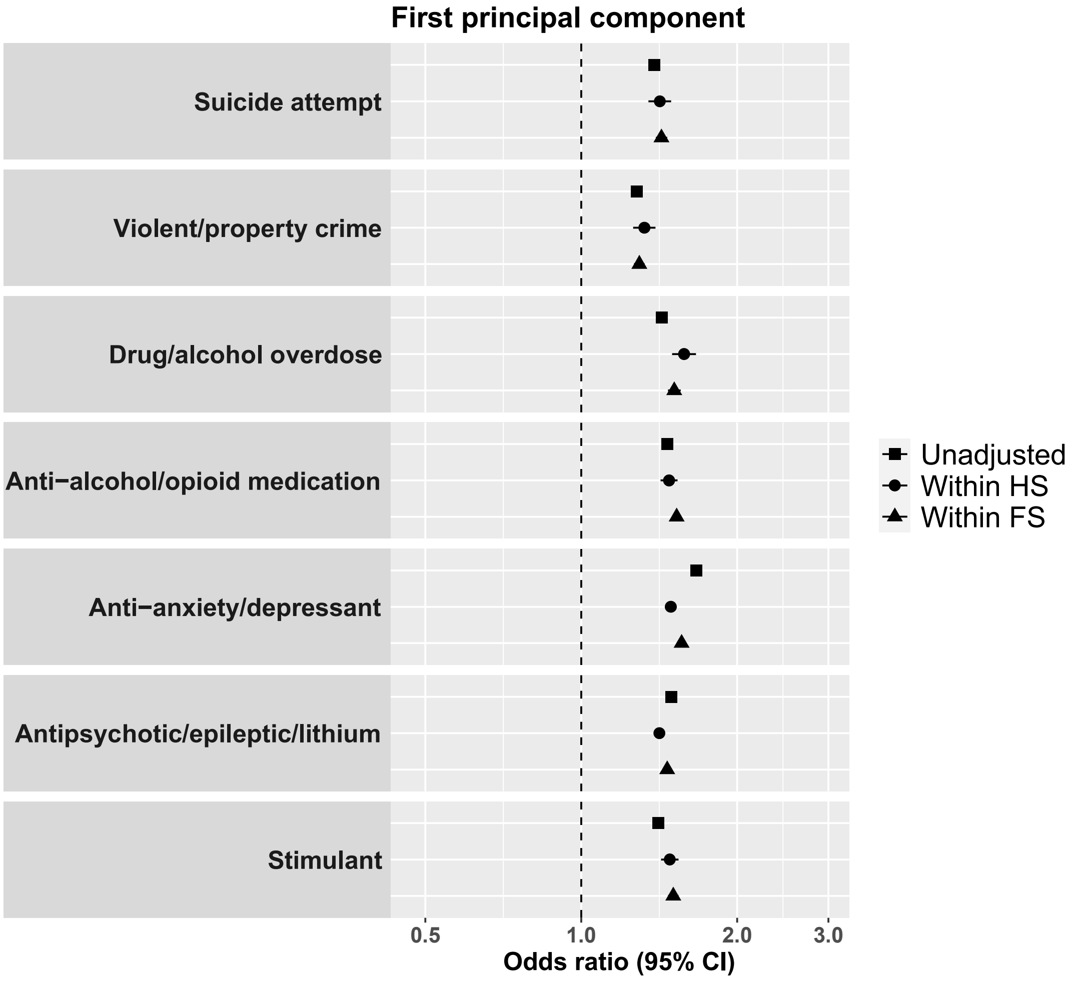

© 2023 Pettersson E et al. *JAMA Psychiatry.*
