# Supplementary material for: Associations Between General and Specific Psychopathology Factors and 10-Year Clinically Relevant Outcomes in Adult Swedish Twins and Siblings
Source: JAMA Psychiatry. 2023 May 10;80(7):728–37. doi: 10.1001/jamapsychiatry.2023.1162 (PMC10173102; doi:10.1001/jamapsychiatry.2023.1162)
Supplement: Supplement 2. — Data sharing statement [file jamapsychiatry-e231162-s002.pdf]

Data Sharing Statement

Pettersson. Associations Between General and Specific Psychopathology Factors and 10-Year Clinically Relevant Outcomes in Adult Swedish Twins and Siblings. *JAMA Psychiatry*. Published May 10, 2023. doi:10.1001/jamapsychiatry.2023.1162

Data
Data available: No

Additional Information
Explanation for why data not available: We do not have consent the share data. However, data from the Swedish population can be applied for at the Swedish National Board of Health and Welfare. Data from the Swedish Twin Studies of Adults: Genes and Environment (STAGE) can be applied for at the Swedish Twin Registry.
